# Homogenised daily lake surface water temperature data generated from multiple satellite sensors: A long-term case study of a large sub-Alpine lake

**DOI:** 10.1038/srep31251

**Published:** 2016-08-09

**Authors:** Sajid Pareeth, Nico Salmaso, Rita Adrian, Markus Neteler

**Affiliations:** 1 Department of Biodiversity and Molecular Ecology, Research and Innovation Centre, Fondazione Edmund Mach, Trento, 38010, Italy; 2 Department of Sustainable Agro-Ecosystems and Bioresources, Research and Innovation Centre, Fondazione Edmund Mach, Trento, 38010, Italy; 3 Freie Universität Berlin, Department of Biology, Chemistry and Pharmacy, Berlin, 14195, Germany; 4 Leibniz Institute of Freshwater Ecology and Inland Fisheries (IGB), Berlin, 12587, Germany; 5 Mundialis GmbH & Co. KG, Bonn, 53111, Germany

## Abstract

Availability of remotely sensed multi-spectral images since the 1980’s, which cover three decades of voluminous data could help researchers to study the changing dynamics of bio-physical characteristics of land and water. In this study, we introduce a new methodology to develop homogenised Lake Surface Water Temperature (LSWT) from multiple polar orbiting satellites. Precisely, we developed homogenised 1 km daily LSWT maps covering the last 30 years (1986 to 2015) combining data from 13 satellites. We used a split-window technique to derive LSWT from brightness temperatures and a modified diurnal temperature cycle model to homogenise data which were acquired between 8:00 to 17:00 UTC. Gaps in the temporal LSWT data due to the presence of clouds were filled by applying Harmonic ANalysis of Time Series (HANTS). The satellite derived LSWT maps were validated based on long-term monthly *in-situ* bulk temperature measurements in Lake Garda, the largest lake in Italy. We found the satellite derived homogenised LSWT being significantly correlated to *in-situ* data. The new LSWT time series showed a significant annual rate of increase of 0.020 °C yr^−1^ (*P < 0.05), and of 0.036 °C yr^−1^ (***P < 0.001) during summer.

Lakes are considered worldwide as sentinels of climate change^1^,^2^. Any change in the surrounding catchment due to climate forcings will reflect on the physical, chemical and biological processes in lakes^2^. The surface temperature as being direct and sensitive to long-term changes in thermal structure of the lakes is a good indicator to understand the changes in the lake characteristics^1^,^3^. Thermal variations on the lake surface and epilimnion are crucial for key responses like vertical mixing and stratification, nutrient and oxygen dynamics, as well as spread and geographical expansion of biota^1^,^2^,^4^. Recent studies on a large number of lakes worldwide indicated a global trend of rapid warming related to climate change^5^,^6^,^7^,^8^. Global warming has a direct impact on thermal characteristics of lakes, influencing variations in the physical as well as biological characteristics^9^,^10^. Geographical expansion of toxic cyanobacteria is reported owing to the warming of lakes^11^,^12^,^13^,^14^. The expansion of tropical bloom-forming cyanobacterium *Cylindrospermopsis raciborskii* (Woloszynska) to the temperate lakes in the mid latitudes is triggered by global warming^15^. The study “Blooms like it hot”^12^ explain climate change as a potent catalyst for the expansion of toxic blooms. In Sweden, annual phytoplankton bloom in larger lakes was found to be anticipated by a month due to the early warming in spring^16^.

Lake Surface Water Temperature (LSWT) exhibits a rapid and direct response to climate forcing prominently induced by changes in air temperature, cloud cover, short wave radiation, latitude, and the lakes morphometry^5^. Detailed synthesis of *in-situ* and satellite derived LSWT over lakes globally, indicated rapid warming during summer. Moreover, LSWT and air temperature trends during summer are found to diverge over many lakes globally which signifies the need of regional studies in understanding the change dynamics^5^. Investigations carried out on the large lakes south of the Alps using non-parametric tests on volume weighted spring mean temperature derived from long-term *in-situ* data series from 1970 to 2009 showed a significant warming rate of 0.012–0.028 °C yr^−1^, at a rate comparable with that of other European and North American lakes^17^,^18^. The year-to-year fluctuations in the thermal structure were shown to be strictly controlled by large scale atmospheric dynamics between the N-Atlantic and the Mediterranean regions^19^^,^^20^. Results of limnological studies carried out in the largest and deepest lakes south of the Alps Lake Garda, Lake Iseo, Lake Como, Lake Lugano and Lake Maggiore 17, pointed out the need of integrating interdisciplinary approaches to the scientific based long-term monitoring. Lack of long-term *in-situ* data at a high temporal frequency is the main obstacle in identifying long-term trends. In this context, data from remote sensing as a substitute to *in-situ* data could play a key role in limnological studies.

Surface temperature is one of the accurate and reliable measurement using remote sensing^10^. Remote sensing of the temperature is based on recording the emitted radiation from earth surface in the spectral domain of 8–14 μm^21^. Inverse Planck’s law is applied to convert the emitted radiance recorded in the thermal infrared region to Top Of Atmosphere (TOA) Brightness Temperatures (BT). There are multiple approaches to estimate land/water surface temperature from the brightness temperatures^22^. In the case of inland water bodies like large lakes, the most common approach is the split-window technique where the difference between the two adjacent thermal channels (10.5–11.5 μm, 11.5–12.5 μm) is taken as a measure of atmospheric attenuation to derive the Surface Temperature (ST)^23^. The accuracy of the derived ST depends on the split-window coefficients, which in turn is based upon multiple parameters like spectral response function and emissivity of the channels, column water vapour in the atmosphere and View Zenith Angle (VZA) of the sensor. These coefficients are derived by regressing simulated BT’s from radiative transfer models like MODTRAN against the atmospheric profiles. The coefficients are generally derived on a regional or global level for the estimation of Land Surface Temperature (LST) and Sea Surface Temperature (SST)^24^,^25^,^26^. For lakes, both LST and SST based algorithms are used interchangeably and with higher accuracy of RMSE between 0.5–1.5 °C^6^,^18^,^27^. Furthermore, lake and sensor specific constants are published for an exhaustive list of global lakes by^31^.

There are a multitude of LST and SST datasets derived from multiple sensors publicly available such as Pathfinder from AVHRR data^32^, EuroLST^33^, GlobTemperature from ATSR series^34^ and the MODIS temperature products. ArcLakes^35^ and the LSWT data by^30^ are two lake specific products derived exclusively from satellite data for a set of selected lakes. A global database of lake summer surface water temperatures for 291 lakes combining both *in-situ* and satellite data was recently published by^36^. Nevertheless, the products are often based on selected sensors aboard multiple instruments with different specifications and acquisition times. The algorithm behind surface temperature retrieval also differ among the products. In addition, many temperature products from moderate resolution (~1 km) sensors are resampled to coarser resolution (~5 km) which results in missing spatial coverage or even complete data loss at lake locations. Since multiple datasets cover different time frames, we need to combine them in order to generate a time series appropriate for trend analysis in the context of climate impact studies. But combining these data from multiple sources without applying acquisition time correction and homogenisation would result in spurious trends.

Our aim was to develop a new methodology to derive daily homogenised LSWT data from multiple moderate resolution sensors. As a result, we developed a new homogenised LSWT time series for Lake Garda using the dual thermal channels from the sensors - AVHRR/2 (NOAA-9/11/12/14), AVHRR/3 (NOAA-16/17/18/19), ATSR1 (ERS-1), ATSR2 (ERS-2), AATSR (Envisat) and MODIS (Aqua/Terra). The new time series offers 30 years of daily LSWT from 1986 to 2015 developed using split-window algorithm with satellite specific coefficients published by^26^, acquisition time/orbital drift corrected, spatially sampled at 1 km resolution and gap filled using harmonic analysis. To our knowledge, this is the first time a homogenised LSWT was developed merging data from 13 satellites. The new method is reproducible and extensible to other lakes globally, provided that satellite data is available. The entire methodology is implemented using open source software packages. Hereafter, in the following sections “AVHRR” represents both AVHRR/2 and AVHRR/3 sensors and “ATSR series” represents ATSR1, ATSR2 and AATSR unless explicitly specified.

## Study area

**Figure 1:**
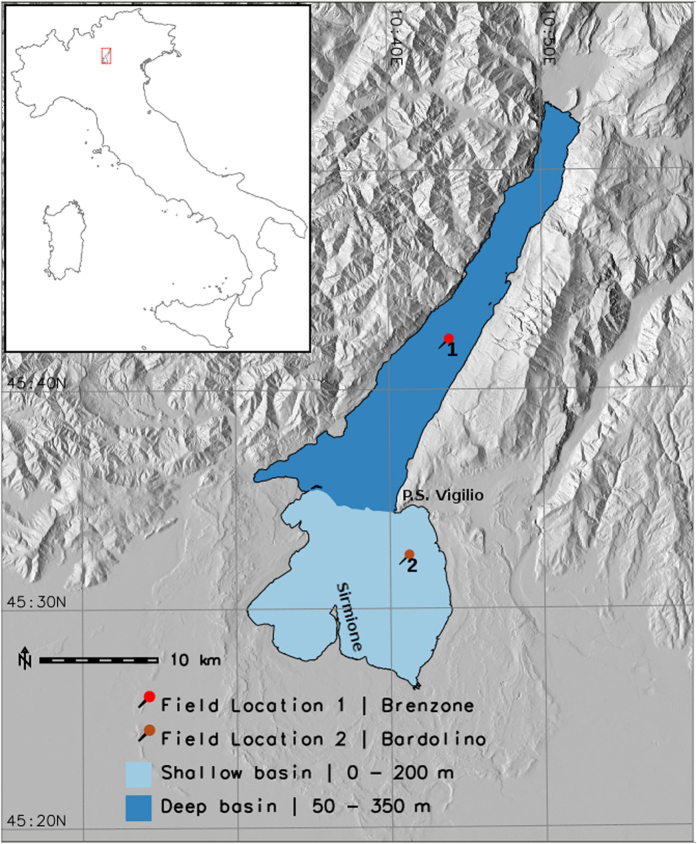
Study area map of Lake Garda; dark blue area depicts the deep basin and light blue area depicts the shallow basin. Push pins represent locations of *in-situ* water temperature monitoring. The maps were generated using the software GRASS GIS 7.0^48^ (URL - https://grass.osgeo.org/grass7/).

Lake Garda is located east of the southern sub-Alpine region between the coordinates - Longitude: 45.44°*E*–45.92°*E*; Latitude: 10.44°*N*–10.91°*N*. With a surface of 368 km^2^ and a volume of 49 km^3^, it is the largest lake in Italy. Along with the lakes Iseo, Como, Maggiore and Lugano it forms part of the group of deep lakes located south of the Alpine chain. These lakes are an important economic resource in Europe. Besides representing a major tourist destination, their waters are also used for drinking water purposes. Lake Garda is divided into two basins, northwestern and southeastern, which are divided by an underwater ridge connecting the Sirmione peninsula with Punta S. Vigilio (Fig. 1). The northwestern basin is large and deep, the shores descend at sharp slopes and the bottom spreads over 20 km at depths from 300 to 350 m (maximum depth). The maximum depth in the southeastern basin is around 80 m, whereas the shape is nearly conical. From a practical point of view, the lake can, however, be divided into two major zones separating the deeper northern area from the shallower southern area (mostly <150 m). This distinction is consistent with the observations in ref. 37 where, using temporal multi-spectral satellite images, found significant spatial and temporal variability in the concentrations of chlorophyll-*a* in the northern and southern areas of Lake Garda. The low ratio between the surface of the catchment area and the surface of the lake (6.1) explains the long theoretical water renewal time of about 27 years in comparison with other deep lakes in the area.

## Results

**Figure 2:**
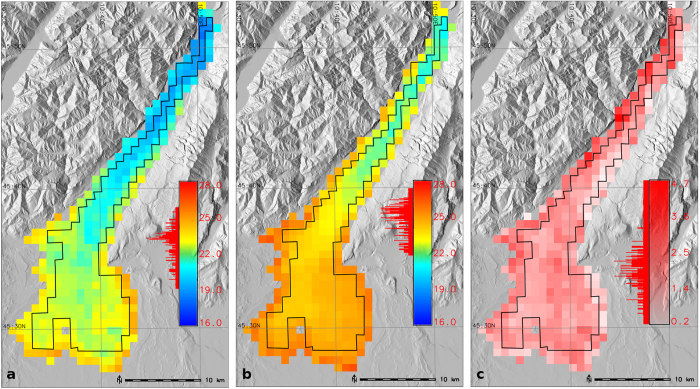
(**a**) Summer mean LSWT of year 1992 (**b**) summer mean LSWT of year 2003 (**c**) difference map between summer means of 2003 and 1992. The boundary layer shown in black over Lake Garda is the inner buffer used to mask out the edge pixels. The maps were generated using the software GRASS GIS 7.0^48^ (URL - https://grass.osgeo.org/grass7/).

**Figure 3:**
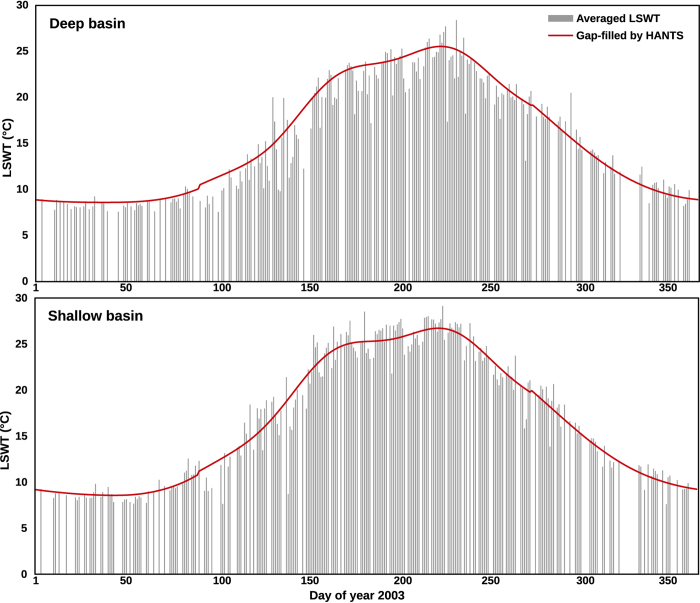
Plots showing gap-filled reconstructed LSWT using HANTS (red line) over the averaged homogenised LSWT (grey bars) from multiple observations over a particular day of the year. The daily LSWT time series from the deep and shallow basins for the year 2003 is shown as an example.

We processed a total of 62,799 level-1B input images in order to derive daily LSWT’s. A single LSWT map has 363 pixels of 1 km resolution representing the entire Lake Garda. After applying the inner mask to remove the edge pixels, a total of 223 pixels were considered for deriving long-term climatologies (Fig. 2). The aggregated seasonal and annual maps were developed by averaging the daily data. To demonstrate the spatial variability of the new LSWT maps, Fig. 2 shows the aggregated summer mean temperature maps for the years 1992 and 2003, along with a temperature difference map. The year 1992 had a relatively cool summer, while the year 2003 had a record warm summer due to a heatwave all across Europe. Harmonic analysis filled data gaps by repetitively applying a least square fit and removing the outliers until the remaining data was within the valid range. The amplitude of the first harmonic in all the years was larger than the other harmonics depicting a strong unimodal pattern of LSWT. To demonstrate the data reconstruction using HANTS, Fig. 3 shows the smoothed LSWT plotted over the daily averaged LSWT after homogenisation for the year 2003.

### Validation

**Figure 4:**
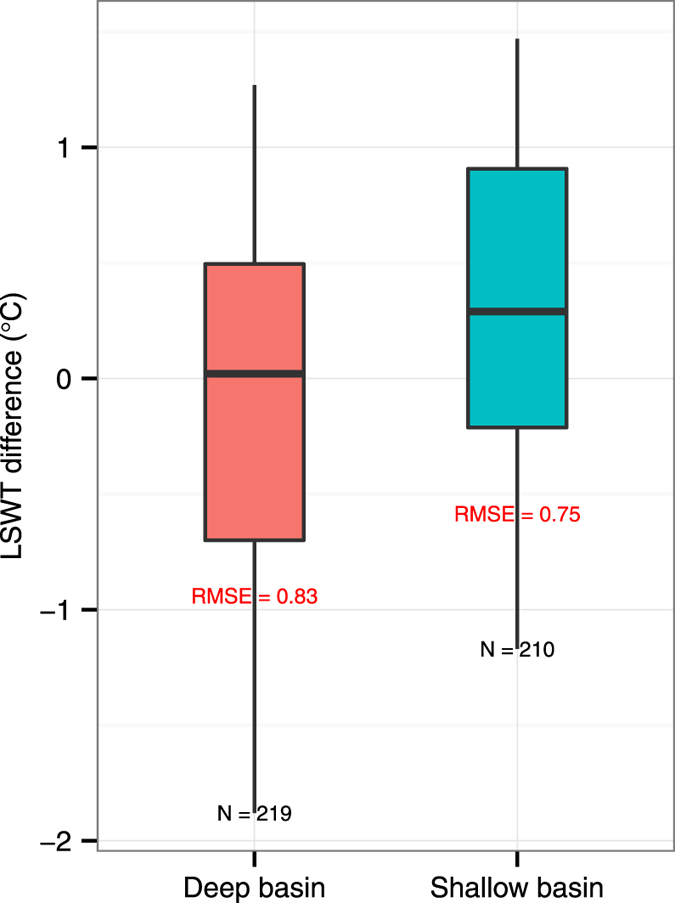
Boxplots of lake mean LSWT difference between final homogenised LSWT and corresponding *in-situ* data. The values shown in black over the lower whisker represents N and the values in red is the reported RMSE in °C.

To validate the new LSWT data, we performed a cross platform comparison of same day observations between the satellite pairs, followed by regression analysis between final homogenised LSWT and the *in-situ* data. The RMSE of the various cross-platform pairs for both deep and shallow basins varied between 0.38 °C and 1.28 °C (Table 1). The minimum RMSE of 0.38 °C was reported for the satellite pair NOAA18/NOAA19 for both the deep and shallow basins. The highest RMSE of 1.2 °C was reported for the pair NOAA17/Terra in the deep basin and of 1.28 °C for NOAA14/Terra in the shallow basin. An average RMSE of 0.88 °C was reported for all the satellite pairs taking into consideration both the basins. Furthermore, we validated LSWT derived from individual satellites with the corresponding *in-situ* data. We were only able to use data from 11 satellites for the validation due to the non-availability of matching *in-situ* data against NOAA9 and NOAA12 (Table 2). An average RMSE of 0.86 °C and 0.94 °C was obtained for the deep and shallow basins respectively. For the deep basin, LSWT derived from NOAA16 had the lowest RMSE against *in-situ* data (0.42 °C), while NOAA18 had the highest RMSE (1.29 °C). In the shallow basin, the lowest RMSE was reported for ERS-2 (0.67 °C), whereas the highest RMSE was reported for Aqua against *in-situ* data (1.18 °C). An average RMSE of 0.92 °C was estimated between satellite derived LSWT and *in-situ* observations. The regression analysis between the final homogenised LSWT and the *in-situ* data revealed a coefficient of determination (R^2^) of 0.98 for both basins. The mean RMSE estimations were 0.83 °C and 0.75 °C for deep and shallow basins respectively (Fig. 4).

### Long term warming trends

**Figure 5:**
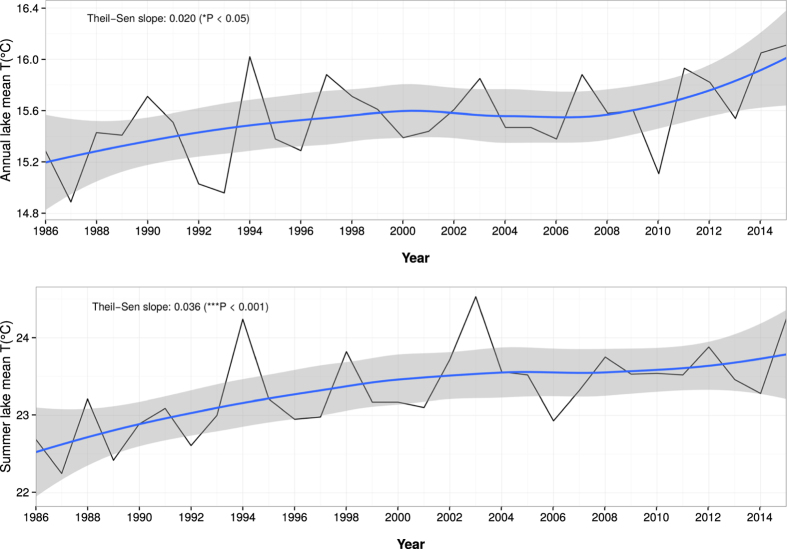
Annual (top) and summer (bottom) trends of lake mean LSWT derived from the new homogenised daily LSWT between 1986 to 2015. Data were smoothed using the loess interpolation (blue line). The gray area is the 95% confidence interval.

**Figure 6:**
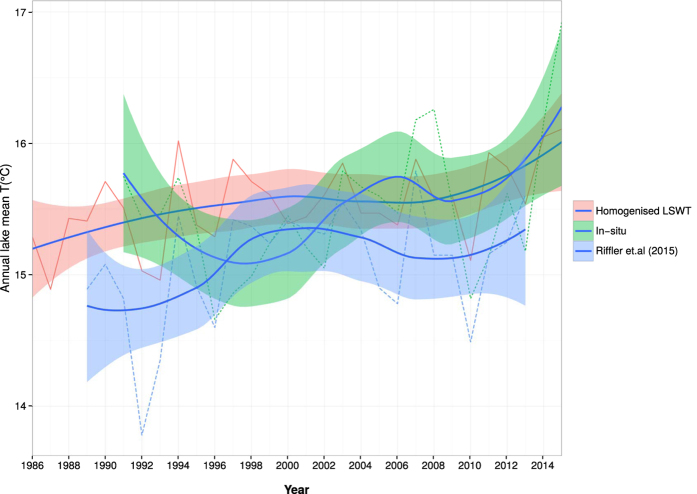
Annual trends of lake mean LSWT computed from homogenised LSWT (deep basin), *in-situ* data and satellite derived LSWT^30^. Data is smoothed using the loess interpolation (blue line). The area shown around the smoothed line is the 95% confidence interval.

We found a significant (*P < 0.05) warming trend for lake mean LSWT at an annual rate of 0.020 °C yr^−1^ (Fig. 5). For the deep basin, we found a similar warming trend (0.014 °C yr^−1^; *P < 0.05) for annual mean LSWT, but for shallow basin there was no significant annual trend. For summer months, we report a significant warming trend at the rate of 0.036 °C yr^−1^ (***P < 0.001) for the lake mean LSWT (Fig. 5). We report a similar increasing rate for the mean LSWT over the deep basin (0.039 °C yr^−1^; ***P < 0.001), while no significant warming was found for the shallow basin. We did not observed any significant trends during the other seasons. Furthermore, standard deviation of 0.32 °C and 0.53 °C were estimated from the time series of annual and summer mean LSWT respectively. For comparison, we performed summer and annual trend analysis on the lake mean temperature computed from *in-situ* data and recently published satellite derived LSWT by^30^. We obtained a significant warming trend at the rate of 0.039 °C yr^−1^ (*P < 0.05) and 0.027 °C yr^−1^ (*P < 0.05) for both summer and annual time series from the data by^30^, while no significant warming was found from the *in-situ* data (Fig. 6).

## Discussion

**Figure 7:**
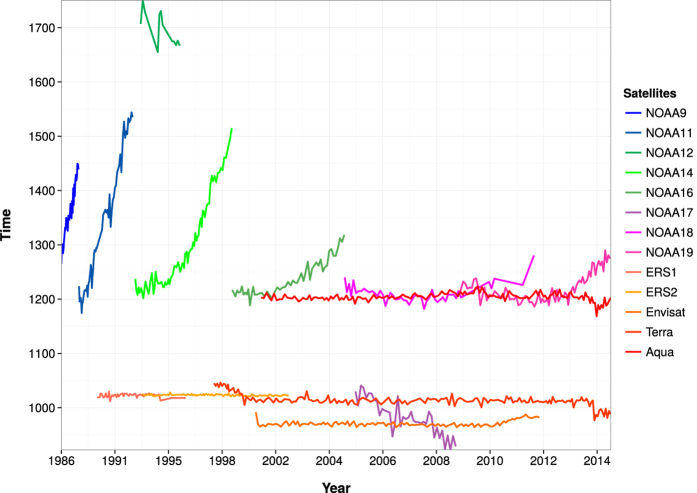
Plot of variation in observation times of all the satellites used in this study. Note the large orbital drifts of the earlier NOAA-9/11/12/14 instruments.

Remotely sensed satellite data offer a great alternative to *in-situ* data. We demonstrated the usability of satellite derived LSWT in estimating long-term annual and seasonal trends of lake mean LSWT. The inevitable breaks in historical satellite data due to different life periods and the overpass time of multiple satellites is the main challenge in developing a continuous time series (Fig. 7). In order to make a long-term time series of any bio-geophysical parameter from satellite data, it is necessary to proceed with a combination of different time series, which demand homogenisation to correct for the different acquisition times. Hence, we developed a workflow to derive daily LSWT maps of Lake Garda for the last 30 years (1986–2015) at 1 km spatial resolution recorded by six moderate resolution sensors on board of 13 different satellites. For AVHRR data acquired from multiple NOAA instruments, we used the calibration techniques which take into account the inter-satellite calibration^38^. For the ATSR series, the calibration coefficients provided with the data were of superior quality ensuring continuity of data with transition of the instrument^6^. Moreover, the split-window technique with satellite specific coefficients used in this study could derive LSWT’s at an average RMSE of 0.88 °C on a single day.

We found similar RMSE (<1 °C) before and after applying time correction procedures between satellite derived LSWT and corresponding *in-situ* data. This could be due to the fact that lake surface temperature of a deep sub-Alpine lake is a slow changing parameter during the day. Precisely, the temperature measured at different times during the day was close enough to exhibit any noticeable change in accuracy indicators before and after the time correction procedure. Nevertheless, for the older satellites, like earlier NOAA instruments which underwent considerable orbital shifts during their course in orbit, this step was crucial. We argue that the acquisition time correction is necessary while combining data from different satellites due to at least two reasons: i) to merge the data obtained from multiple satellites which have different quality, acquisition times and orbital decays; ii) for more dynamic surfaces where temperature varies at short temporal scales, this step may prove to be crucial. Moreover, identical slopes from different linear models between individual satellites and *in-situ* data confirmed the comparability of different satellite derived LSWT to each other.

Harmonic analysis was used to remove the gaps in the daily data due to undetected clouds and unresolved spurious calibration. We found that the gap filled daily data was over smoothed which could inadvertently remove some of the actual temporal dynamic change. In spite of this smoothing, the final homogenised LSWT data was able to pick up the long-term thermal dynamics over the lake surface. From the long-term summer trend of LSWT (Fig. 5), it was evident that the new LSWT data was able to pick up the low and high peaks in the last 30 years. Moreover, the spatial coverage of 1 km is another advantage of this dataset in comparing the thermal dynamics of different locations in the lake. The temperature difference map in Fig. 2 clearly illustrates how LSWT is distributed over the surface of Lake Garda, whereby the shallow basin is more warm than the deep basin. Figure 2 also shows that in the deep basin, the western shores are warmer than at the eastern side. This is explained by the high mountain chain of Mt. Baldo (2218 m) in the east, which blocks the sun for a long time during the day. The amplitude and phase maps obtained for each harmonic per year provide additional information on lake’s thermal variation over time. The amplitude of the first harmonic is the highest for all the years, showing the influence of annual cycle of LSWT on the lake characteristics.

We performed validation at different stages of the work flow before and after homogenisation. We found that the RMSE obtained between individual satellite derived LSWT and *in-situ* data varied significantly between the deep and the shallow basins (Table 2). The higher RMSE in the shallow basin may be due to the varying skin effects in Lake Garda. The difference between skin and bulk temperature often termed as *skin effect* could be a deciding factor in varying accuracy over the deep and the shallow basins^6^,^27^,^41^. It must be noted that, the satellite measured LSWT represents temperature of a sub-micron layer between the lake surface and the air, and is highly variable according to the meteorological conditions. The *in-situ* data on the other hand represents bulk temperature, often measured between 0 to 0.5 m. The RMSE values were closer in the cross-platform comparison analysis in both the deep and shallow basins (Table 1), which demonstrates the superior quality of split-window coefficients. With respect to the sensor performance in deriving LSWT, we found that the ATSR series exhibited lower RMSE of 0.75 °C and 0.74 °C, followed by AVHRR with RMSE of 0.88 °C and 0.96 °C, MODIS with RMSE of 0.92 °C and 1.18 °C for deep and shallow basins respectively. A similar study by^30^ also reported RMSE between 1 to 1.6 °C for AVHRR derived LSWT against *in-situ* data from multiple European lakes. For a better comparison it is important to have high frequency *in-situ* data matching the exact acquisition time of the satellites^6^,^29^. The majority of the LSWT difference in both basins were below 1.0 °C. This suggests that the homogenisation procedure follwed by HANTS retained the same pattern of accuracy and the RMSE in a similar range. Moreover, the comparison between summer lake mean trends obtained from our new LSWT data series and a similar satellite derived LSWT by^30^ showed that both the data series are comparable and exhibits similar trends (Fig. 6).

To understand the long-term dynamics of lake surface temperature, it is crucial to have high frequency *in-situ* observations. In this study, we demonstrate that the satellite derived data can complement the missing *in-situ* observations of surface water temperature, with high spatial and temporal resolution. Global coverage of the satellite data is an added advantage which enables us to use the same data set for analyzing multiple lakes subsequently for comparative analyses. The correct evaluation of the long-term trends of water temperature should take into account the advantages and limits of measurements made by both remote sensing techniques and *in-situ* recordings. Remote sensing technologies are limited to the detection of long-term trends circumscribed to the surface of water bodies. While this limit can be compensated by measurements performed at high temporal (daily) and spatial (whole lake) scales, the direct measurement of surface data in the field at lower temporal frequency can seriously impair the evaluation of long-term trends. Conversely, the lower temporal and spatial coverage of *in-situ* data can be partly balanced by a complete availability of data collected in the water column. The thermal structure of the mixolimnetic layer is less vulnerable to transient (daily to weekly scales) fluctuations of surface water temperatures originating from changes in local meteorological conditions. Therefore, most of the studies which have focused on the long-term trends of lake water temperatures have been based on measurements recorded in the water column. Lake Garda being a deep sub-Alpine lake, undergo complex mixing processes during winter and spring which have a strong influence on surface water temperatures^20^. The inter-annual change of LSWT could be helpful in identifying impacts on the associated physical and biological processes in the lake.

Recent studies by^18^ and^5^ have confirmed global warming of lakes due to climate change between 1985 and 2009 at the rate of 0.03–0.04 °C yr^−1^. The main difference between the two studies is that^18^, used exclusively the satellite derived LSWT, while^5^ used a combination of *in-situ* and satellite data to derive the long-term trends. There are regional studies showing rapid warming of North American large lakes and European lakes which uses satellite derived LSWT^6^,^30^. With a different approach^7^,^17^, and^42^ reported warming at similar rates using *in-situ* volume weighted mean bulk temperature for the large lakes in Europe and North America. In contrast to our approach, many earlier studies used LST and SST data for lake studies which reported similar results. More recently, studies by^30^ and^31^ developed lake specific surface temperature data using optimized split window coefficients. We used the data by^30^ for comparing the trends and obtained similar increasing temperature trends during summer. Study by^1^ reported July warming since 1970 at the rate of 0.02–0.05 °C yr^−1^ computed from *in-situ* data for multiple northern hemisphere lakes. Although we did our analysis separately for the deep and shallow basins of Lake Garda, we could not find any significant trend in the shallower, southern basin. Moreover, the thermal variation over the deep basin was similar to the entire lake basin, depicting that the lake is more influenced by the characteristics of the deep basin.

One of the main advantages of the developed method is that it gives the opportunity to study the long-term thermal dynamics of other lakes given the availability of the satellite data over study area. The spatial and temporal coverage obtained from this method is unique and cannot be achieved by any other data source. The outliers due to undetected clouds and other unsuitable data, are often difficult to remove completely. Though the method is robust, manually checking the accuracy of cloud masks for thousands of images is not practical. The automated cloud masking may leave undetected cloud pixels as clear sky ones. A future enhancement for refining cloud masks would be to use seasonal thresholds in SPARC algorithm (see Methods) instead of global ones. Nevertheless in this study, HANTS were able to remove the outliers as shown by the validation results at the expense of losing some of the short term temporal variability. The spatial resolution of 1 km is the highest possible when considering moderate resolution satellite data, but it is not high enough to study the smaller lakes. Moreover at this resolution, the issue with mixed pixels along the shore were solved by using an inner lake buffer while computing long-term climatologies.

## Methods

### Data

**Figure 8:**
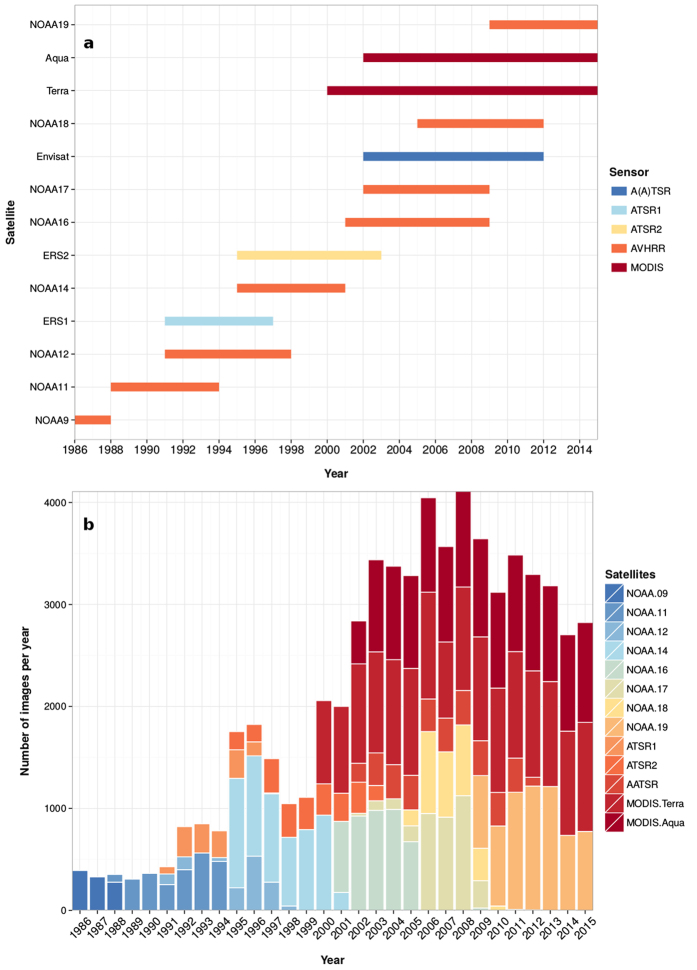
Availability of moderate resolution satellite data between 1986 and 2015. (**a**) Time line of the satellites in orbit; (**b**) Distribution of level-1B data downloaded for the study area from 1986 to 2015 obtained from respective archives - CLASS (AVHRR), LAADS (MODIS) and Merci (ATSR series) separated by different instruments.

In this study we used multiple moderate resolution sensor data to estimate long-term LSWT’s over the past 30 years. We used data acquired by six sensors - AVHRR/2, AVHRR/3, ATSR1, ATSR2, A(A)TSR and MODIS which were on board 13 satellites (Fig. 8a). These sensors offer dual thermal channels at 10.5–11.5 μm and 11.5–12.5 μm with a spatial resolution of approximately 1 km. This was ideal for deriving surface water temperature using a split-window algorithm. The sensors AVHRR and MODIS provide daily images, while ATSR series provide data every three days. We downloaded and processed level-1B data of AVHRR, ATSR series and MODIS from Comprehensive Large Array-data Stewardship System (CLASS - http://www.class.ncdc.noaa.gov/), Merci data archive (http://ats-merci-ds.eo.esa.int/merci/welcome.do) and Level 1 and Atmosphere Archive and Distribution System (LAADS - https://ladsweb.nascom.nasa.gov/) respectively for the study area. For AVHRR, we used Local Area Coverage (LAC) data available at its original spatial resolution of 1.1 km. For ATSR series, we downloaded and processed nadir viewing TOA data. The level-1B swath products MYD021KM and MOD021KM for Aqua and Terra satellites respectively were used to extract dual thermal channels from MODIS sensor. Figure 8b shows the year wise distribution of data from multiple satellites. We processed the AVHRR and MODIS data using Pytroll libraries (http://www.pytroll.org/). Furthermore, we used BEAM software provided by European Space Agency to process the ATSR series of data (http://www.brockmann-consult.de/cms/web/beam/). For validation of the satellite derived water temperatures, long-term monthly *in-situ* data between 1991–2015 collected from two locations in Lake Garda were used. We used bulk temperature (0–0.5 m) measured at two locations - Brenzone in the deep basin and Bardolino in the shallow basin (see Fig. 1).

### Pre-processing

The pre-processing of thermal channels at level-1B include thermal calibration to convert from radiances to brightness temperatures using inverse Planck’s law followed by removal of cloud pixels. The data from MODIS and ATSR series were calibrated using the coefficients provided in the header. For AVHRR, the calibration procedure was much more complicated due to (well documented) navigational errors with the older NOAA satellites^43^. Despite on board thermal calibration in NOAA instruments, the calibration data were prone to solar contamination and atmospheric attenuation of the signal and satellite decays^40^. In this study, we used the geometrically corrected time series of brightness temperatures derived from dual thermal channels of AVHRR LAC data from 1986 to 2015 developed by^44^. Furthermore, we considered only those pixels acquired at a zenith angle less than 45 degrees. All the further data processing was restricted to lake pixels masked using a Lake Garda boundary layer. For the cloud masks of level-1B AVHRR LAC and MODIS data, we used the Separation of Pixels Using Aggregated Rating over Canada (SPARC) algorithm developed by^45^, originally implemented for creating cloud mask over Canada. We used two relevant tests to remove thick clouds and thin cirrus above the lakes, i) brightness temperature test (T-test) using channel T_i_ and ii) thin cirrus test (C-test) which uses the difference between channel T_i_ and T_j_. The T-test uses channel T_i_ brightness temperature and compares it with a dynamic threshold which is the surface skin temperature data of the corresponding day and time derived from climatic models. In this study, we replaced the North American regional reanalysis data with European centre for medium-range weather forecasts^46^ following the successful implementation over Europe by^30^. The SPARC algorithm is implemented using raster processing tools in GRASS GIS 7.0^47^,^48^. For ATSR series, we used the quality layer provided with the data to remove the cloud pixels.

### Deriving LSWT using split-window algorithm

From the brightness temperatures derived by thermal calibration, the next step was to estimate LSWT’s using split-window algorithm. We used a non-linear split-window Equation 1 to estimate the surface temperature as proposed by^26^:


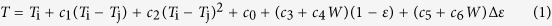


where c_0_–c_6_ are split-window coefficients, T_i_ and T_j_ are at-sensor brightness temperatures derived from the dual thermal channels, *ε* is mean emissivity, Δ*ε* is emissivity difference and W is the total atmospheric water vapour column (g/cm^2^). For water surface, the mean emissivity is close to unity. As our study is dealing with lakes, the equation 1 was modified by assuming *ε* as one and Δ*ε* to be zero. Hence the modified equation 2 was used in this study to retrieve LSWT from dual thermal channels:


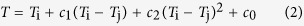


We used satellite specific split-window coefficients published by^26^. These coefficients were derived by statistical minimization from a simulated database of brightness temperatures and atmospheric profiles obtained from MODTRAN radiative transfer model, emissivity spectra extracted from spectral libraries and the spectral response functions^26^.

After estimating the LSWT, we applied two levels of filters to remove outliers due to undetected clouds and spurious calibrations. First, we applied a global filter based on a minimum and maximum thresholds derived from the long-term *in-situ* data. Thus we discarded any LSWT values beyond the range 6.5–29 °C. We further applied an advanced filter based on inter-quartile range of LSWT’s derived every 16 days^33^,^49^. The lower and upper thresholds for this filter was computed using the equations 3 and 4 respectively:


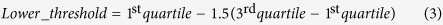



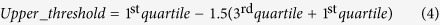


where the 1^st^quartile and 3^rd^ quartile were derived climatologically from the LSWT data every 16 days over the years 1986 to 2015. This filter was then applied using the derived thresholds to those LSWT data of corresponding time frames.

### Homogenising LSWT from multiple satellites

**Figure 9:**
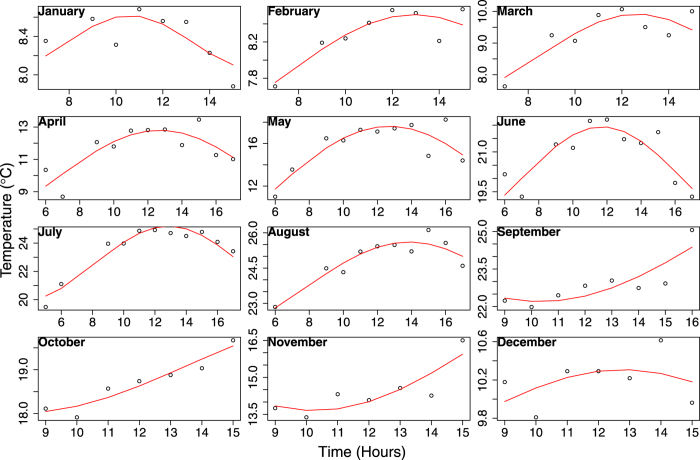
Monthly diurnal cycles during day time estimated using long-term hourly climatologies from satellite observations. The points in plot are the long-term hourly averages for a particular month, and the red line is DTC model fit.

We considered all the satellite observations for a single day over a wide window of time; from 08:00 to 17:00 UTC. Figure 7 clearly shows the orbital drift of earlier satellites - NOAA-9/11/12/14/16. We applied a physical model based on typical pattern technique proposed by^50^ to standardize the acquisition time to 12:00 UTC. In this approach, monthly diurnal cycles were used as a reference pattern to correct for the varying observation times to produce a standard observation time. The monthly diurnal cycles computed from hourly averages were derived from the existing satellite observations of LSWT and were limited to day time (Fig. 9). We followed the Diurnal Temperature Cycle (DTC) model to derive the diurnal variations of LSWT based on^51^. The model used was described by the Equation 6:


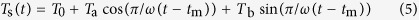


with


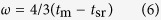


where T_0_ is the residual temperature at sun rise; T_a_ and T_b_ are temperature amplitudes; T_s_(t) is surface temperature at time *t*; t_m_ is the time at which temperature is maximum; t_sr_ is the time of sun rise; *ω* is calculated using the Equation 6. T_0_, T_a_ and T_b_ are obtained by statistically fitting the model given in the Equation 5. Finally, to apply the time correction to all the LSWT data, for each image with an observation time *t*, T_s_(t) and T_s_(12) were calculated using the model as given in Equation 5. The correction factor was then computed using Equation 7 to correct the LSWT to a standard time at 12:00 UTC. A condition based approach is used for the correction as given in Equation 8 and 9:


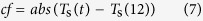



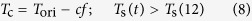



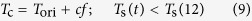


where *cf* is the correction factor, T_c_ is the corrected LSWT to a standard time 12:00 UTC and T_ori_ is the actual LSWT.

In order to derive one homogenised LSWT per day, the subsequent step was to merge by averaging the single day observations from multiple satellites. For this, we first performed a satellite specific linear regression analysis between LSWT and *in-situ* data to compare the slope of regression lines between different satellites. We performed the analysis on the deep and the shallow basins separately. The slopes varied between 0.85 and 1.1, depicting that the data used for the models were similar to each other and exhibited similar residual patterns. We used the *r.mapcalc* module in GRASS GIS 7.0 software to apply the time correction model to the LSWT data.

### Gap filling using harmonic analysis

The daily LSWT data after time correction and averaging over the same day still suffered from gaps due to cloud cover. We used Harmonic ANalysis of Time Series (HANTS) to reconstruct the gaps in the LSWT data. The harmonic analysis decomposes a time series into several sinusoidal functions with unique amplitude and phase values^52^. It applies a least square fitting procedure based on the derived harmonics^53^. The fitting procedure is repeated until the LSWT values with large deviations are removed from the fitted curve^54^. Hence, the role of HANTS is twofold in this process; i) to remove the outliers, and ii) fill the gaps with fitted values. The fitting procedure based on HANTS is explained well by^53^ and^54^. We implemented HANTS based reconstruction on LSWT images using the *r.hants* addon in GRASS GIS 7.0 software^55^.

### Temporal database and trend analysis

The gap filled daily LSWT maps from 1986 to 2015 were then imported into a spatio-temporal database using TGRASS modules in GRASS GIS 7.0. TGRASS is an advanced set of modules which perform time series analysis on spatio-temporal data^56^. We developed annual and seasonal climatologies from the daily LSWT data to analyse the long-term trends. The seasonal climatologies were developed by aggregating the LSWT over four seasons; winter (December/January/February), spring (March/April/May/), summer (June/July/August) and fall (September/October/November). We applied non-parametric tests on annual and seasonal mean LSWT separately for deep and shallow basins to detect the long-term trends. In particular, we used the Mann-Kendall test to identify the presence of monotonic upward or downward trends. We computed the Theil-Sen slope for the quantitative estimation of significant trends^57^. We used the Durbin-Watson test to detect potential serial correlation issues in the time series and checked for its statistical significance. To avoid mixed pixels with land along the shore, we used an inner lake buffer considering only water pixels while extracting the annual and seasonal climatologies (Fig. 2). Furthermore, for comparative analysis, we computed the same statistics on long-term *in-situ* data and satellite derived LSWT from AVHRR sensor by^30^ for Lake Garda.

## Additional Information

**How to cite this article**: Pareeth, S. *et al.* Homogenised daily lake surface water temperature data generated from multiple satellite sensors: A long-term case study of a large sub-Alpine lake. *Sci. Rep.* **6**, 31251; doi: 10.1038/srep31251 (2016).

## Figures and Tables

**Table 1:** RMSE in °C reported between same day observations from different pair of satellites for the deep and shallow lake basin.

Satellite pair	RMSE (Deep basin)	RMSE (Shallow basin)
NOAA17/NOAA18	0.74(71)	0.71(85)
NOAA18/NOAA19	0.45(10)	0.38(9)
NOAA11/ERS-1	0.95(37)	0.91(37)
NOAA14/ERS-1	0.78(19)	0.82(16)
NOAA14/ERS-2	0.78(90)	0.89(90)
NOAA16/ERS-2	0.76(38)	0.93(37)
NOAA16/Envisat	0.85(58)	0.87(50)
NOAA17/Envisat	0.67(26)	0.57(29)
NOAA18/Envisat	0.73(84)	0.76(95)
NOAA19/Envisat	0.66(75)	0.75(78)
ERS-2/Envisat	0.79(37)	0.51(33)
NOAA14/Terra	1.19(23)	1.28(22)
NOAA12/NOAA14	1.11(22)	1.05(20)
NOAA16/Terra	1.11(147)	1.17(139)
NOAA17/Terra	1.20(68)	1.16(76)
NOAA18/Terra	0.98(205)	1.02(220)
NOAA19/Terra	1.07(323)	1.12(310)
ERS-2/Terra	0.94(55)	0.87(56)
Envisat/Terra	1.18(212)	1.05(243)
NOAA16/Aqua	0.97(138)	0.97(130)
NOAA17/Aqua	1.06(63)	0.99(70)
NOAA18/Aqua	0.82(252)	0.73(256)
NOAA19/Aqua	0.97(397)	0.86(380)
ERS-2/Aqua	0.99(20)	0.78(20)
Envisat/Aqua	1.10(215)	0.98(211)
Terra/Aqua	0.78(858)	0.86(809)

Number inside brackets represents N - Number of observations.

**Table 2:** RMSE in °C reported at deep basin and shallow basin from absolute difference between *in-situ* data and satellite derived LSWT.

Satellites	RMSE (Deep basin)	RMSE (Shallow basin)
NOAA11	0.33(9)	0.93(9)
NOAA14	0.54(14)	0.51(17)
NOAA16	0.33(8)	0.91(8)
NOAA17	0.37(4)	0.86(3)
NOAA18	1.25(11)	0.90(13)
NOAA19	0.67(16)	1.14(14)
ERS-1	0.41(5)	0.74(4)
ERS-2	0.47(14)	0.70(12)
Envisat	0.50(10)	0.50(11)
Terra	0.92(41)	1.04(36)
Aqua	0.89(46)	1.17(38)

Number inside brackets refer to N - the number of same day observations.
